# Cerebral Amyloidoma Resulting from Central Nervous System Lymphoplasmacytic Lymphoma: A Case Report and Literature Review

**DOI:** 10.1155/2018/5083234

**Published:** 2018-06-26

**Authors:** Geetha Jagannathan, Guldeep Uppal, Kevin Judy, Mark T. Curtis

**Affiliations:** ^1^Department of Pathology, Thomas Jefferson University Hospital, Philadelphia, PA 19107, USA; ^2^Department of Neurosurgery, Thomas Jefferson University Hospital, Philadelphia, PA 19107, USA

## Abstract

Cerebral amyloidomas are rare cerebral mass lesions often associated with significant morbidity. Cerebral amyloid accumulation can be the result of a number of disease states and it is crucial for proper patient care to identify the pathogenic process leading to amyloidoma formation. Low grade clonal B-cell processes are one cause of cerebral amyloidomas. We report a case of an 87-year-old woman who presented with a lymphoplasmacytic lymphoma associated cerebral amyloidoma complicated by cerebral hemorrhage, discuss the proper workup of this disease entity, and present a review of the literature on this topic.

## 1. Introduction

Cerebral amyloidomas are uncommon cerebral mass lesions that prior to biopsy are often thought to be glial neoplasms, high grade lymphomas, or immune mediated processes. Following identification of a brain mass as amyloidoma, the etiology of the massive central nervous system (CNS) amyloid deposition must still be determined. Causes of CNS amyloidomas include clonal CNS B-cells, cerebral amyloid angiopathy (CAA), and inherited amyloidopathies. We report a case of an 87-year-old with a biopsy diagnosed cerebral amyloidoma, discuss the appropriate workup to identify the underlying disease cause and present a literature review of cases of B-cell associated CNS amyloidomas, and raise the possibility that patients with cerebral amyloidomas may be at risk for cerebral hemorrhages.

## 2. Case Report

### 2.1. Case History

**Figure 1:**
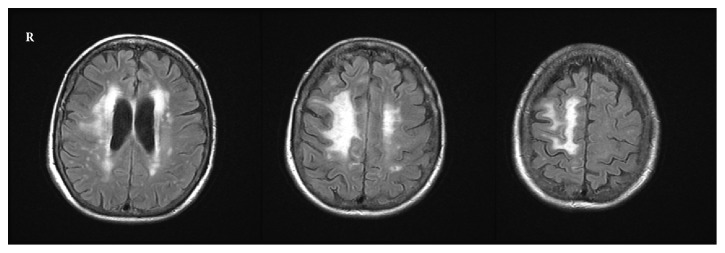
MRI with contrast shows multifocal T2 hyperintense abnormalities with the largest focus in the right frontal lobe.

A previously well 87-year-old Caucasian woman living in a senior assisted care center presented to the neurology clinic with complaints of six months of slowly progressing left sided weakness. Initial difficulty in ambulating and using the stairs progressed to being wheelchair bound. Neurologic exam revealed diffuse 3/5 left sided weakness, left leg drift, and left facial droop. Brain magnetic resonance imaging (MRI) revealed a large confluent white matter T2-hyperintensity in the right frontal lobe with multifocal nodular enhancement of the left cerebral hemisphere (Figure 1). Foci of enhancement were also identified in the cerebellum and leptomeninges. The radiologic differential diagnosis included vasculitis, lymphoma, and CNS sarcoidosis as the most probable causes of the multifocal disease process, with glial neoplasm, demyelination, and metastases considered less likely.

### 2.2. Histopathology and Lab Findings

All sample analysis described below were performed on material obtained by brain biopsy as part of clinical care. All samples were obtained with appropriate consent.

**Figure 2:**
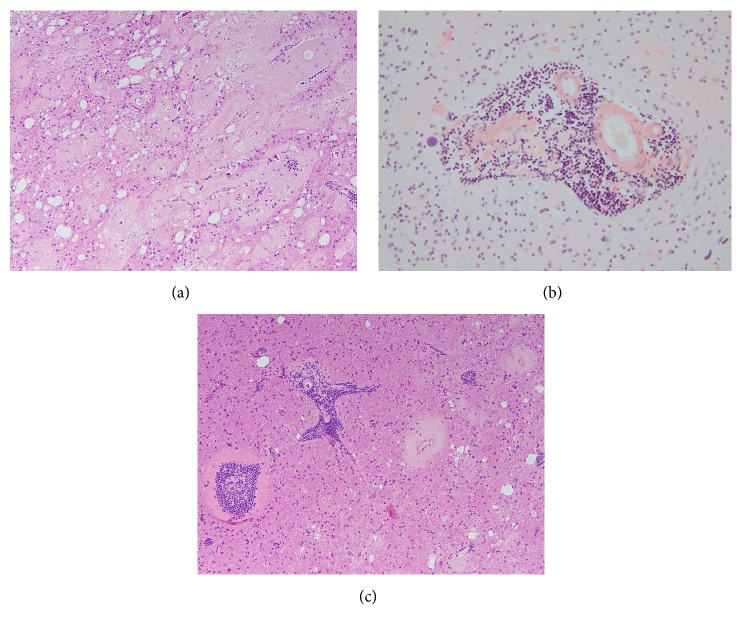
(a) Abundant accumulation of parenchymal, vascular, and perivascular amyloid, hematoxylin and eosin stain, original magnification 100x. (b) Congo-red stain highlights the amyloid deposition in a parenchymal blood vessel that also shows perivascular lymphocytic infiltration, original magnification 200x. (c) Small intraparenchymal blood vessels with perivascular lymphoplasmacytic infiltrate, hematoxylin and eosin stain, original magnification 100x.

A biopsy of the mass was performed and revealed extensive parenchymal lakes and vascular and perivascular deposition of amorphous, amyloid like material (Figure 2(a)). Congo-red positive staining and apple-green birefringence (not shown) of the amorphous material upon polarization confirmed that the amorphous material was amyloid (Figure 2(b)). Also present in the resected tissue were a number of small intraparenchymal blood vessels with perivascular lymphoplasmacytic infiltrates (Figure 2(c)). The initial histologic differential diagnoses included cerebral amyloid angiopathy-inflammatory type (CAA-I) and lymphoma associated amyloidoma. To identify the underlying etiology of the amyloid accumulation, a number of additional analyses were performed.

**Figure 3:**
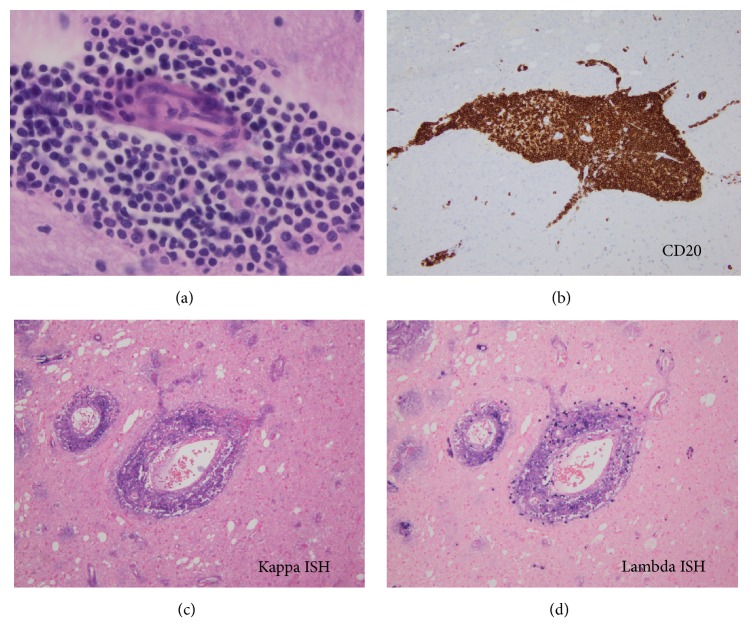
(a) Perivascular infiltrate highlighting the lymphoplasmacytic appearance of the cells, hematoxylin and eosin stain, original magnification 600x. (b) Immunohistochemical stain for CD20 highlights a perivascular infiltrate of B-cells, original magnification 200x. (c) and (d) Positive staining of the tumor cells for lambda light chains but not for kappa light chains signifies lambda light chain restriction,* in situ *hybridization, original magnification 200X.

Liquid chromatography tandem mass spectroscopic analysis identified the amyloid as AL *λ*-type and not *β* amyloid or an amyloid associated with a hereditary amyloidosis. Further analysis of the perivascular lymphoid populations was undertaken. Histologically, the monotonous populations of perivascular lymphoid cells demonstrated a lymphoplasmacytic appearance (Figure 3(a)). Immunohistochemical analysis demonstrated that the lymphoid cells were CD20 positive (Figure 3(b)). Tumor cells were negative for CD3, CD5, BCL1, and CD23. The tumor Ki67 proliferation index was low (3%). The more plasmacytoid appearing cells were CD138 positive and were shown to be lambda light chain restricted by kappa and lambda chromogenic* in situ* analysis (Figures 3(c) and 3(d)). An immunoglobulin heavy chain (IgH) gene rearrangement analysis of the brain tissue from this case was positive for a clonal process with a 253-base pair peak in the FR2 region. A MYD88 L265P mutation analysis by PCR-based pyrosequencing on the brain tissue from this case was negative. A diagnosis of a low grade, lymphoplasmacytic lymphoma (LPL) was rendered. The identification of this CNS low grade lymphoplasmacytic lymphoma confirmed the cause of the amyloidoma to be a lambda light chain producing lymphoplasmacytic lymphoma.

To determine if an extracranial/systemic lymphoplasmacytic lymphoma was the source of the CNS neoplasm, a bone marrow biopsy was performed. The bone marrow biopsy showed normal trilineage hematopoiesis and no evidence of lymphoma, myeloma, or amyloidosis. Cytogenetics and fluorescent* in situ *hybridization studies on the bone marrow were negative for genetic aberrations. Urine protein and serum immunoglobulin levels were within normal limits. A biopsy of subcutaneous abdominal adipose tissue was negative for amyloid, demonstrating lack of evidence of systemic amyloid deposition. Interestingly, an IgH gene rearrangement analysis on the bone marrow was positive for a clonal gene rearrangement with two peaks: a 282-base pair peak in FR2 region and a 120-base pair peak in FR3 region in a polyclonal background, which importantly were markedly different from the IgH gene rearrangement identified in the CNS lymphoplasmacytic lymphoma. Since the two-small bone marrow clonal peaks are present in a polyclonal background, their significance is uncertain and may be age related.

### 2.3. Treatment and Clinical Outcome

Our patient received one cycle of chemotherapy with Rituximab for Primary CNS lymphoplasmacytic lymphoma. Two months after diagnosis, she developed a hemorrhagic infarct on the left frontal white matter and was transferred to hospice care.

## 3. Discussion

Our case represents a massive accumulation of lambda light chain amyloid (lambda AL amyloidoma) resulting from a primary CNS lymphoplasmacytic lymphoma. Lymphomas confined to the brain, primary central nervous system lymphomas (PCNSL), are rare and account for only 2-3% of all brain tumors and <1% of all non-Hodgkin's lymphoma (NHL). A recent epidemiological review of the data from 10 Surveillance, Epidemiology, and End Results (SEER) cancer registries in the United States between 1992 and 2011 estimated that 90% of PCNLs are B-cell lymphomas, of which the vast majority (75%) are aggressive diffuse large B-cell lymphomas. Primary brain involvement by less aggressive small mature B-cell lymphomas is even more rare. Close to 40% of PCNSL are associated with immunosuppression, in the form of either human immunodeficiency virus (HIV) infection or drug induced immunosuppression. No immunodeficiency was identified in the patient in this case. Over the last few decades the incidence of primary central nervous system (CNS) lymphomas has however shown a significant increase in immunocompetent patients [4].

The amyloidoma in this case was caused by a CNS low grade lymphoplasmacytic lymphoma. Low grade small B-cell lymphomas can have overlapping morphological and immunophenotypic features and varying degrees of plasmacytic differentiation that make their diagnosis challenging. Typically, LPL involves the bone marrow but may also involve other sites such as spleen and lymph nodes. The majority of LPL produce IgM monoclonal paraprotein, which is also known as Waldenström macroglobulinemia and may cause systemic hyperviscosity related symptoms. Rarely, systemic LPL may involve the central nervous system, a condition known as Bing Neel syndrome. In our case, no systemic LPL was identified. Recently, Xu et al. reported the presence of MYD88 (L265P) mutations in approximately 90% IgM paraprotein producing LPLs [5]. Genetic aberrations that are commonly described in the systemic small mature B-cell lymphomas are however less frequently observed in their primary CNS counterparts. MYD88 mutation analysis on our case of primary CNS LPL was negative. The lack of identification of the MYD88 (L265P) mutation in the lymphoplasmacytic lymphoma in the case reported here may indicate that this tumor is one of the MYD88 mutation negative LPLs or possibly that the quantity of neoplastic LPL cells was insufficient for detection of the mutation. Future MYD88 analysis of CNS LPLs will help to address this issue.

Monoclonal immunoglobulin deposition in the brain, like elsewhere in the body, can either be in the form of light chain deposition disease (LCDD) or amyloidosis. In LCDD, the light chain deposits are characterized by less structural organization and do not stain positive on Congo-red stain. In amyloidosis on the other hand, the light chains form compact beta pleated sheet predominant structures that stain positive for Congo-red and show a characteristic apple-green birefringence on polarization [6]. Amyloidosis of the brain is not uncommon and can be seen associated with *β*-amyloid accumulation in Alzheimer's disease and cerebral amyloid angiopathy. However, primary CNS involvement by AL subtype are less common. It is important to note that CNS involvement by systemic amyloidosis seldom occurs due to the extremely ineffective passage of systemic amyloid across the blood brain barrier [7].

To gain further insight into the occurrence of low grade B-cell lymphomas and their association with the formation of CNS amyloidomas, we performed a literature search of the PubMed database for primary CNS small low grade B-cell lymphoma. Our search identified 12 cases of primary CNS lymphoplasmacytic lymphoma [2, 8–10], one large case series with 69 cases of primary CNS marginal zone lymphoma (MZL) [11], one case of primary CNS follicular lymphoma [12], and one case of primary CNS small lymphocytic lymphoma (SLL) [13]. Among these cases, we found 5 cases of primary CNS low grade small B-cell lymphomas that presented in association with intracerebral AL amyloid deposition [Table 1]. Three cases were marginal zone lymphomas and two (including our case) were lymphoplasmacytic lymphomas. Recently, He*β* et al. demonstrated clonality in cases of CNS AL amyloidomas with background nonspecific inflammatory infiltrates by chromogenic* in situ* hybridization (CISH) for kappa and lambda light chains and IgH rearrangement studies [14]. AL amyloidomas of the CNS appear to arise either in the setting of a plasmacytoma or a low grade lymphoma with a plasmacytic differentiation and often the underlying hematologic dyscrasia is not obvious. These lesions present as focal neurological deficits or seizures. Primary CNS MZL are commonly dural based [11].

There is no clear treatment modality of choice for AL amyloidomas with low grade lymphomas of the CNS, and the cases reported have been treated with surgery, radiation, and chemotherapy either in isolation or in combination. Irrespective of treatment method, these low grade lymphomas tend to behave in an indolent manner. One patient [Table 1] relapsed but did well with change of chemotherapy. As described above, the patient described in this report received one cycle of chemotherapy with Rituximab and, however, then developed a left frontal lobe hemorrhagic infarct. While these lesions may behave in an indolent fashion, cerebral hemorrhage in the setting of a brain amyloidoma has been previously reported [15]. Beta-amyloid associated cerebral amyloid angiopathy is known to be a cause of cerebral hemorrhages. Vascular amyloid involvement in AL amyloidomas may also predispose to vascular events.

In conclusion, workup of a cerebral amyloidoma should include studies to characterize the nature of the amyloid and if indicated to identify a clonal/neoplastic B-cell population. If a B-cell neoplasm is identified, treatment of the underlying B-cell tumor is indicated. Increased surveillance for hemorrhagic vascular infarcts is warranted given the association of vascular amyloid deposition and a risk of cerebral hemorrhage.

## Figures and Tables

**Table 1a:** (a) Presentation, treatment, and outcome of primary CNS mature B-cell lymphomas presented as an amyloidomas published in literature

**Reference**	**Age/sex**	**Symptoms at presentation**	**Duration of symptoms**	**site**	**Clinical diagnosis**	**Treatment**	**Follow-up**	**Diagnosis rendered**
(1) Lehman et al. [1]	63F	Focal sensory seizure- trigeminal neuralgia and mild right sided hearing loss	3 years	Dural, frontal lobe	Meningioma	Surgical resection of the largest mass, radiation therapy	8 months- alive with disease, imaging showed no change in the size of the amyloid mass	Primary CNS Marginal zone lymphoma

(2) Pace et al. [2]	46F	Seizures	Acute onset	Left frontal lobe	Oligodendroglioma or metastasis	Surgical resection only	24 months- no evidence of disease	Primary CNS lymphoplasmacytic lymphoma

(3) Tu et al. [3]	49 M	Seizures	NR	Dural, left frontal	NR	Methotrexate, recurrence treated with fludarabine	7.6 years- Recurrence at 4 months, No evidence of disease after treatment with fludarabine	Primary CNS Marginal zone lymphoma

(4) Tu et al. [3]	62F	Ataxia	NR	Dural, left occipital	NR	Radiation	25 months- No evidence of disease	Primary CNS Marginal zone lymphoma

(5) Our case	87F	Left sided weakness	6 months	Right frontal lobe	Lymphoma, vasculitis, or sarcoidosis	Rituximab, status post 1st cycle	2 months- patient developed hemorrhagic stroke	Low grade B-cell lymphoma with plasmacytic differentiation

NR: not reported; ND: not done.

**Table 1b:** (b) Diagnostic testing performed on the primary CNS mature B-cell lymphomas presented as an amyloidomas published in literature

**Reference**	**Imaging**	**Histopathology**	**Light chain restriction**	**Bone marrow**	**Systemic amyloidosis**	**Lab tests**	**Molecular testing**
(1) Lehman et al. [1]	MRI- isointense on T1 and hyperintense on T2	Intracerebral and vascular amyloid deposition, CD 20+ B cells and plasmacytoid cell infiltrates, CD138+ plasma cells	Kappa	Normal	NR	SPEP and UPEP- normal, CBC- normal	ND

(2) Pace et al. [2]	MRI- isointense on T1, hypointense on T2	Intracerebral and vascular amyloid deposition, CD 20+ B cells and plasmacytoid cell infiltrates, CD138+ plasma cells	Kappa	Normal	Ruled out by serum amyloid P component scan	SPEP- normal, normal serum free kappa, free lambda and kappa: lambda ratio	ND

(3) Tu et al. [3]	NR	Amyloid deposition, CD20 + B cell infiltrates	Kappa	NR	NR	NR	Trisomy 3 positive, Negative for MALT1 and IgH translocation

(4) Tu et al. [3]	NR	Amyloid deposition, cyclin D1-, CD20 + B-cell infiltrates	Kappa	NR	NR	NR	Trisomy 3 positive, Negative for MALT1 and IgH translocation

(5) Our case	MRI-T2 hyperintense	Intraparenchymal and perivascular amyloid deposition (AL subtype). CD20+, CD3-, CD5-, BCL1-, CD23- plasmacytoid lymphocytes and CD138+ plasma cells	Lambda	Normal	Ruled out by abdominal fat biopsy	Normal serum free kappa, free lambda and kappa: lambda ratio	MYD88 L265P mutation analysis was negative

NR: not reported; ND: not done.
